# Application of Biochar on Soil Improvement and Speciation Transformation of Heavy Metal in Constructed Wetland

**DOI:** 10.3390/biology14050515

**Published:** 2025-05-07

**Authors:** Yuan Zhou, Xiaoqin Nie, Yao Zhao, Liqiu Zhang, Yatian Cheng, Cancan Jiang, Wenbin Zhao, Xiangchun Wang, Chao Yang

**Affiliations:** 1 Innovation Center for Ecology and Landscape Architecture Engineering Technology, Ministry of Housing and Urban-Rural Development, Beijing 100120, China; zhouyuan@cucd.cn (Y.Z.); wangxiangchun@cucd.cn (X.W.);; 2 China Urban Construction Design & Research Institute Co., Ltd., Beijing 100120, China; 3 School of Marxism, Beijing Forestry University, Beijing 100083, China; 4 Beijing Key Laboratory for Source Control Technology of Water Pollution, College of Environmental Science and Engineering, Beijing Forestry University, Beijing 100083, China; 5 Engineering Research Center for Water Pollution Source Control & Eco-Remediation, College of Environmental Science and Engineering, Beijing Forestry University, Beijing 100083, China; 6 School of Architecture, Tsinghua University, Beijing 100084, China; 7 Key Laboratory of Environmental Biotechnology, Research Center for Eco-Environmental Sciences, Chinese Academy of Sciences, Beijing 100085, China

**Keywords:** sewage sludge, biochar, Pb contamination, soil remediation, Pb speciation transformation

## Abstract

The pyrolysis of sewage sludge into biochar, enhanced by incorporating agriculture waste rich in inorganic minerals and lignocellulosic compounds, provides an effective approach for achieving sludge-harmless treatment and resourceful utilization. In this study, sewage sludge and maize straw-based biochar (SMB) was prepared using the co-pyrolysis method, and the effects of different application ratios (0%, 1%, 3%, and 5%, *w*/*w*) of SMB on soil properties, ryegrass growth, microbial community structure, and Pb content and speciation in the contaminated soil of constructed wetlands were investigated. The results showed that SMB had a high carbon content (28.58%) and was rich in functional groups (e.g., -C-O, -C-N). The results indicated that increasing SMB dosage (0–5% *w*/*w*) in Pb-contaminated soil elevated soil pH from 6.40 to 7.93, cation-exchange capacity (CEC) from 30.59 to 79.03 cmol/kg (+158%), and organic carbon content by 65% (from 176.79 mg/kg to 107.3 mg/kg), while reducing available phosphorus and potassium by 20% and 30%, respectively, resulting in a 6% decline in ryegrass leaf length. SMB application enriched Pb-resistant bacteria (e.g., *Sphingomonas* abundance increased from 10.3% to 11.2%) and enhanced Pb immobilization. After 55 days, the total soil Pb increased by 33%, and the residual fraction Pb significantly increased by 7.3% to 21.7%, driven by functional group complexation, ion exchange, pH, and CEC improvements.

## Simple Summary

This study developed biochar from co-pyrolyzed sewage sludge and agricultural waste, combining inorganic and organic components to stabilize lead in contaminated soils. The biochar enhanced soil properties and immobilized lead through chemical and microbial mechanisms, prioritizing long-term contamination control despite minor plant growth trade-offs. By transforming waste into a soil amendment, this approach innovatively addresses sewage sludge disposal and heavy metal pollution simultaneously, advancing sustainable strategies for circular agriculture and eco-remediation.

## 1. Introduction

With the rapid development of urbanization and increasing wastewater production, large amounts of sewage sludge have been generated from wastewater treatment plants. Sewage sludge consists of various organic matter, inorganic particles, heavy metals, pathogens, and other toxic substances [1,2]. Traditional methods of sewage sludge disposal include incineration, landfilling, and land application [1,3]. However, each of these above methods has shortcomings. For example, incineration releases contaminants such as dioxins, landfilling may cause risks of soil pollution due to the existence of pathogens and heavy metals in the sewage sludge, and land application has limitations in land retention capacity [3]. Therefore, environmentally friendly disposal technology for sewage sludge is urgently needed.

Pyrolysis has gained much attention as a promising method for sewage sludge disposal because of its ‘zero waste’ system, and biochar is produced as a solid product during the pyrolysis process [4,5,6]. The pyrolysis of sewage sludge achieves efficient volume reduction (>70%), complete decomposition of volatile organic compounds (VOCs), and pathogen inactivation while simultaneously immobilizing heavy metals (e.g., the heavy metal leaching rate was reduced by 5.5% on average) through stable char-mineral matrix formation [5,7]. Studies have confirmed that the co-pyrolysis of sewage sludge with lignocellulosic biomass, such as maize straw, corncob, shell, and rice husk, could enhance the organic carbon content by 20–35% and reduce heavy metal mobility compared to single sludge pyrolysis. This is attributed to the formation of a biomass-derived carbon matrix, which improves thermal stability and dilutes metal concentrations via ash-organic interactions [8,9,10].

Biochar produced from the co-pyrolysis of sewage sludge and other wastes has been widely applied in soil remediation [6,11,12]. Soil application of biochar can not only stabilize carbon and improve soil properties but also solve the problems of heavy metal pollution and decrease the toxicity of metals in contaminated soil [11,13]. Recent studies have highlighted that biochar derived from the co-pyrolysis of sewage sludge and lignocellulosic feedstocks (e.g., rice husks and maize straw) synergistically enhances soil organic carbon (10–25% increase) and pH (0.5–1.5 unit), while immobilizing heavy metals (e.g., 30–60% Cd/Pb reduction via surface complexation and mineral co-precipitation) and influence plant growth [6,12,14]. Ryegrass is often used as a biological indicator to monitor environmental changes in different habitats, such as lawns, pastures, and constructed wetlands, due to its physiological sensitivity and adaptability [15].

Soil lead (Pb) pollution poses significant environmental risks due to its persistence, bioaccumulation, and toxicity, threatening plant viability and human health via food chain transfer [4,7]. While biochar application has been widely explored for Pb immobilization through mechanisms such as electrostatic interactions, ion exchange, and functional group complexation (e.g., carboxyl and hydroxyl groups), existing studies often oversimplify the interplay between biochar-induced soil property changes and Pb speciation dynamics [9,16]. Critically, the environmental risk of Pb is not only dependent on its total concentration but also on its bioavailability, which is governed by speciation transformations (e.g., exchangeable to residual fraction) modulated by biochar feedstock, soil pH, and amendment dosage [13,14]. However, the systematic quantification of how modified biochar (SMB) dosage regulates Pb speciation and its correlation with soil physicochemical shifts and plant growth (i.e., CEC and organic carbon) remains underexplored. This study uniquely integrated the role of SMB in Pb immobilization and speciation transformation, bridging mechanistic insights with dose dependence to advance precision remediation strategies.

In this study, sewage sludge and maize straw-based biochar (SMB) were prepared using a co-pyrolysis method. The main purposes of this work were as follows: (i) to investigate the variations in soil properties, ryegrass growth, and microbial community structure after SMB application using pot experiments; (ii) to evaluate the effects of SMB on Pb content and speciation transformation in contaminated soil during remediation; and (iii) to assess the optimal SMB proportion suitable for plant growth and soil remediation.

## 2. Materials and Methods

### 2.1. Materials

The sewage sludge used in this study was dewatered sludge (80% water content, *w*/*w*) produced from an anaerobic-anoxic-oxic (A^2^/O) treatment process at the Beijing Gaobeidian Wastewater Treatment Plant. Maize straw was obtained from a rural area in the Changping district of Beijing, China. The feedstocks (sewage sludge and maize straw) were collected and dried for 24 h at 105 °C in a drying oven (101-1 AB, Teste, Tianjin, China), cut into 1–2 cm pieces, and sieved through a 100-mesh sieve. Sandy soil was collected from a suburb in the DaXing district of Beijing. Samples were taken from the top layer (0–200 mm) of soil, air dried, sieved to <0.2 mm, and stored in sealed containers. The characteristics of these raw materials are listed in Table 1. Moisture, ash, volatile matter (VM), fixed carbon (FC), and pH were measured according to Standard Methods [17]. The elementary compositions (C, H, O, and N) of the feedstocks were analyzed using an elemental analyzer (Vario EL cube, Langenselbold, Germany).

### 2.2. Preparation and Characterization of SMB

SMB was prepared by a pyrolysis process at 500 °C for 2 h with a heating rate of 10 °C/min under a continuous flow of N_2_ (purity > 99.9%) and finally cooled to room temperature. Considering the carbon and ash contents of raw materials, sewage sludge, and maize straw were mixed at a ratio of 1:1 (*w*/*w*) for a higher FC content in SMB, according to our previous work [6]. After the co-pyrolysis procedure, the physicochemical characteristics of SMB (pH, elementary analysis, CEC, surface area, and functional groups) were determined.

The pH value of SMB was analyzed by mixing 2 g of SMB with 40 mL of deionized water after 30 min of shaking at 200 rpm, and then the suspension was measured with a pH meter produced by Hach Company (HACH, Loveland, CO, USA). The point of zero charge (PZC) of sludge-modified biochar (SMB) was determined via the pH drift method by equilibrating SMB suspensions (0.1 g/50 mL) in 0.01 M NaNO_3_ under varying initial pH (2–12) for 24 h. The CEC value was estimated using the ammonium ion (NH_4_^+^) exchange method, according to Song et al. [18]. The surface area and surface porosity of the biochar were analyzed at 77 K using an N_2_ adsorption-desorption isotherm by Quantachrome Poremaster 60 (Quantachrome, Boynton Beach, FL, USA). Heavy metal contents (i.e., Pb, Cd, Mn, Zn, Ca, Al, Fe, Mg, and Na) were measured by Inductively Coupled Plasma Mass Spectrometry (ICP-MS) (Agilent 7900, Santa Clara, CA, USA) after microwave digestion using a Mars Microwave Digestion Apparatus (CEM, Matthews, NC, USA) with concentrated HNO_3_ in PFA (Polyfluoroalkoxy) containers at 180 °C (USEPA 3051). Scanning electron microscopy (SEM, Hitachi S-3400N, Naka, Japan) was used to describe the biochar structures. The functional groups were analyzed by Attenuated Total Reflection-Fourier Transform Infrared Spectroscopy (ATR-FTIR) (Bruker Vertex 70, Ettlingen, Germany) at room temperature in the 400–4000 cm^−1^ wave number range.

### 2.3. Pot Experiments

Pot experiments were conducted to evaluate the effects of different proportions of SMB on soil properties, ryegrass growth, microbial community structure, and Pb speciation transformation in constructed wetland soil. According to GB 15618-2018 [19], Pb concentration above 500 mg/kg in soil can create environmental concerns. Soil was polluted according to relevant research [20] by mixing with the prepared Pb(NO_3_)_2_ solution to obtain contaminated soil with a Pb level of 500 mg/kg. This study involved two types of blank soil. The first was uncontaminated control soil, which provided a baseline for soil properties. The second treatment was Pb-contaminated soil without biochar (SMB0). This study mainly explored the effects of biochar on polluted soil before and after application, focusing on comparing the remediation effects on SMB0, which was soil treated with Pb(NO_3_)_2_. Firstly, plastic pots were filled with 5 kg of Pb-contaminated soil, and different proportions of SMB to soil were added at 0%, 1%, 3%, and 5% (*w*/*w*), which were marked as SMB0, SMB1, SMB3, and SMB5, respectively. Each treatment had five completely randomized replicates. Secondly, ryegrass was planted in each pot. Ryegrass was commonly used as an indicator plant for evaluating heavy metal bioavailability during soil remediation [15]. The moisture content of each pot experiment treatment was maintained at 20–30% (*w*/*w*) by adding 100 mL of deionized water every two days. Experiments were conducted at 23 °C and 12 h illumination of fluorescent lamps in the laboratory. The temperature was maintained at about 23 °C throughout the experiment. After 55 days of growth, the fresh weight and leaf length of the ryegrass were measured. The shoots of ryegrass were washed with deionized water and freeze-dried for Pb content analysis. The biochar-amended soil was sampled destructively every five days and then analyzed for soil pH, CEC, contents of total organic carbon in soil (SOC), available N, P, K, microbial community structure, and Pb speciation distribution in soil.

### 2.4. Soil Properties Analysis

Soil organic carbon was measured using the Walkley and Black method (1974). Alkali-hydrolyzable N in the soil was determined using a Kay-type nitrogen determination apparatus by Hanon Instruments (K9840, Jinan, China). Available P content was determined by the NH_4_F-HCl extraction and colorimetry method [19]. Available K was extracted from soil with 1 M ammonium acetate (NH_4_OAc) and measured according to Kongthod et al. [21]. The microbial community structure of the soil was analyzed using bacterial 16S rRNA gene sequences via PCR (polymerase chain reaction) according to a previously reported method [22]. The distribution of Pb speciation in soil was characterized according to the Community Bureau of Reference (BCR) extraction procedures, and specific steps were found in a previous study [1].

### 2.5. Statistics Analysis

The experimental data in this study were calculated using Excel 2010 and analyzed using Origin 9.1. One-way analysis of variance (ANOVA) was used to determine whether there were differences between each treatment. Correlations among different parameters (Pb content, pH, CEC, SOC, ryegrass growth, and microbial community structure) were established using Pearson’s correlation at a significance level of 0.05 using SPSS 13.0.

## 3. Results and Discussion

### 3.1. Properties of Sewage Sludge and Maize Straw

The properties of sewage sludge and maize straw, including ash, VM, FC, and element contents, were measured and are shown in Table 1. It has been recognized that ash is generated from the incomplete combustion of organic and inorganic materials in sewage sludge [6,23]. Compared to the single sewage sludge, the ash content of the biochar significantly decreased when mixed with alternative maize straw. This could be attributed to the lower ash content and higher organic compound contents in maize straw than in sewage sludge. It can be observed that adding maize straw increased the VM and FC contents of feedstocks, which indicated that more organics were present in SMB and were beneficial for soil improvement after SMB application. Higher FC and lower VM contents can also result in a higher yield of biochar during pyrolysis [24].

The heavy metals in sewage sludge mainly included Ca, Al, Pb, Fe, Mg, K, Na, and Zn, and the contents of heavy metals were associated with the source of feedstock (i.e., sludge and maize straw) and the type of flocculants used in the wastewater treatment process. Numerous studies have demonstrated that most heavy metal species in sewage sludge feedstocks can be effectively immobilized under optimized pyrolysis conditions (typically 300 °C–700 °C) and subsequently retained within the biochar matrix during pyrolysis [5,23,24]. Moreover, the leaching concentrations of heavy metals in SMB were tested in Table S1, and the results indicated that they were lower than the thresholds set in GB 5085.3-2007 [25], indicating that SMB could be safely used after pyrolysis.

### 3.2. Properties of SMB

#### 3.2.1. Physicochemical Characteristics of SMB

The characteristics of SMB are also listed in Table 1, including VM, FC, ash content, elemental components, pH, CEC, and heavy metal contents. The co-pyrolysis of sewage sludge and maize straw resulted in an increase in the ash and FC contents in SMB (by 24.91% and 21.88%, respectively), while the VM content in SMB decreased dramatically from 61.52% to 14.65% (*w*/*w*) compared with that in sewage sludge. The increase in ash content was likely attributed to the inherently high mineral and inorganic constituent concentrations in sewage sludge, while the decrease in VM reflected the thermal decomposition of organic components present in both sewage sludge and maize straw during pyrolysis [26]. The increase in FC content indicated that the concentrated and retained carbonate constituents in SMB were improved during pyrolysis compared to those in sewage sludge.

The pH value of SMB increased to alkaline (pH = 8.2) compared with sewage sludge (pH = 6.0), which could be attributed to the release of alkali salts from the feedstock pyrolytic structure and ash component in the SMB surface. The pH drift analysis confirmed that the PZC was aligned with this alkalinity. This alkaline PZC (pH = 8.2 > soil pH) confers a negatively charged SMB surface in typical acidic-to-neutral soils (pH 5–7), enhancing CEC and heavy metal immobilization while synergistically elevating soil pH to promote metal hydroxide precipitation [7]. The decrease in the acidic functional groups in SMB, caused by the oxygen percentage losses, also contributed to the increase in the pH value. The CEC value of SMB reached 29.93 ± 0.55 cmol/kg, which was higher than that of single sewage sludge biochar (25 cmol/kg), representing higher fertilizer maintenance and the buffering ability for soil amendment [18]. It also showed that SMB had a large specific surface area (163.11 m^2^/g), which was much higher than that of single sewage sludge biochar [3]. The H/C ratio is generally considered to represent the charring degree of biochar, and a lower H/C ratio predicts a higher carbonization extent [27]. The lower H/C ratio in SMB (0.13), which was below the stability limit (<0.2) [28], also implied that SMB could be resistant to decomposition and persist in the soil system for many years.

#### 3.2.2. SEM Images of SMB

**Figure 1:**
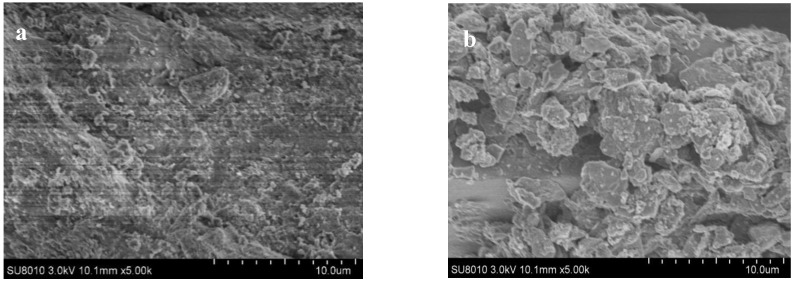
SEM micrographs of sewage sludge (**a**) and SMB (**b**).

SEM was used to investigate the surface microstructure morphology. SEM images of the sewage sludge and SMB are shown in Figure 1. It could be seen that there were obvious differences between sewage sludge and biochar (Figure 1a). In contrast to the smooth surface of sewage sludge with limited porosity and ash deposition, the SMB biochar surface was characterized by a dense and heterogeneous dispersion of ash particles (Figure 1b). This could be explained by the volatilization of organic matter and water-gas shift reactions [6] during pyrolysis, which indicated that SMB has a high surface area for pollutant adsorption in soil remediation [29].

#### 3.2.3. Functional Groups of SMB

**Figure 2:**
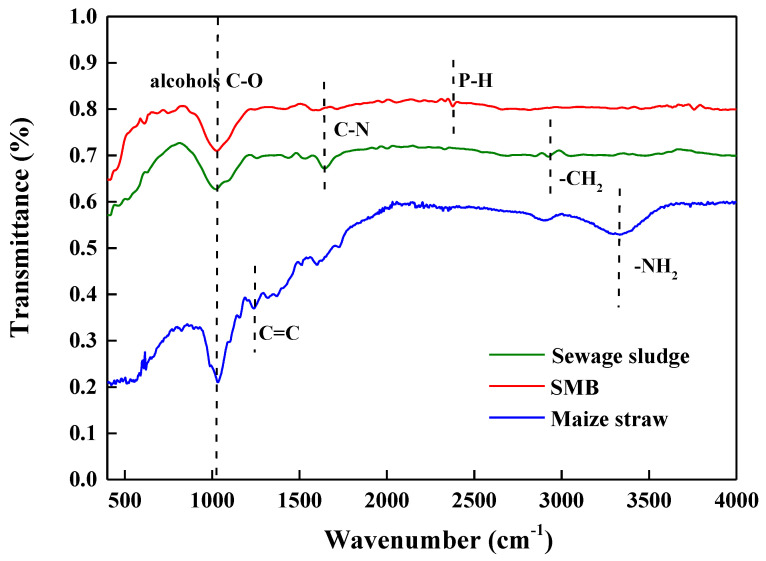
Functional groups of SMB and feedstocks.

The functional groups of SMB and the feedstocks were measured using FTIR, and the results are shown in Figure 2. The broadband near 1033 cm^−1^ was attributed to the C-O functional groups of alcohols. The bands at 1651 cm^−1^ and 2750 cm^−1^ wavelengths were associated with the C-N and P-H stretching bands of SMB, respectively. It could be observed that after co-pyrolysis of sewage sludge and maize straw, bands of alcohols C-O and C-N changed dramatically, indicating the decomposition of organic constituents (e.g., cellulose) and N-containing compounds in the feedstocks [30]. Oxygen and C-N functional groups can provide surface sites for Pb adsorption [31]. At the same time, some functional groups disappeared during pyrolysis, for example, C=C (1250 cm^−1^) and -NH_2_ (3300–3400 cm^−1^) in maize straw and -CH_2_ (2975 cm^−1^) in sludge. The generation of the P-H band suggested an increase in phosphorus during pyrolysis, which could be from sewage sludge and beneficial for Pb adsorption [31].

### 3.3. Influence of SMB on Soil Properties

#### 3.3.1. Influence of SMB on Soil pH and CEC

**Figure 3:**
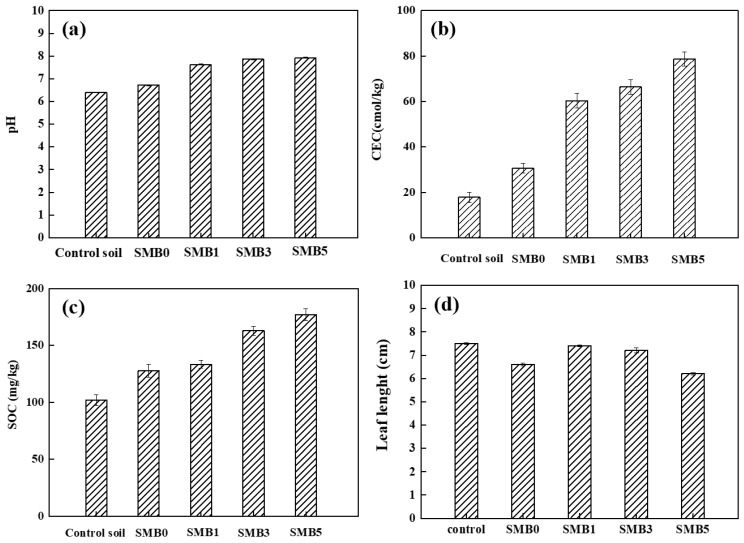
Influence of SMB on soil properties and ryegrass growth ((**a**). soil pH; (**b**). soil CEC; (**c**). SOC; (**d**). leaf length of the ryegrass).

As shown in Figure 3a, variations in pH values in the control soil and Pb-contaminated soil (i.e., SMB0) with different proportions of SMB after 55-d of incubation are presented. The results showed that compared with the control soil (pH = 6.40), the soil pH increased in the Pb-contaminated soil and all biochar-amended soils. The pH values of the soil treated with SMB1, SMB3, and SMB5 increased to 7.63 and 7.86 to 7.93. Studies have also indicated that sewage sludge biochar can increase the pH of acidic soil from 5.0 to 5.6 after biochar application [32]. The increase in pH in the SMB-amended soils was probably explained by the effect of functional groups, alkali salts, soluble carbonates, and metal ions such as K, Ca, Na, and Mg in the prepared SMB, which could effectively neutralize soil acidity [33,34].

CEC value has been used to evaluate the capacity of ion exchange and soil fertility [35]. The influence of SMB application on soil CEC after 55 d of incubation is shown in Figure 3b. It can be seen that soil CEC value gradually increased from 30.59 ± 2.07 to 79.03 ± 2.13 cmol/kg with the increase of SMB proportion from 0 to 5% (*w*/*w*). It is generally accepted that biochar can significantly improve soil CEC, which is mainly attributed to the dissolution of many alkaline substances (e.g., carbonates and metal oxides) in biochar [20]. A high CEC value indicates high soil fertility and a desirable influence on ryegrass growth. Moreover, the elevated CEC of biochar could provide abundant active sites for Pb adsorption through ion exchange while concurrently inducing precipitation via surface complexation, collectively contributing to its immobilization in contaminated soil [16].

#### 3.3.2. Influence of SMB on Soil Organic Carbon and Nutrient Contents

As shown in Figure 3c, the effect of SMB application on the SOC content in Pb-contaminated soil after 55 days was observed. SOC contents in Pb-contaminated soil significantly increased (*p* < 0.05) with an increase in SMB proportion. The highest SOC value was 176.79 ± 4.21 mg/kg when the soil was amended with SMB5. Numerous studies have reported that biochar derived from sewage sludge and agriculture residues can increase soil carbon content [36,37], which can be attributed to the increase in carbonate fractions and soil carbon sequestration after biochar application.

**Figure 4:**
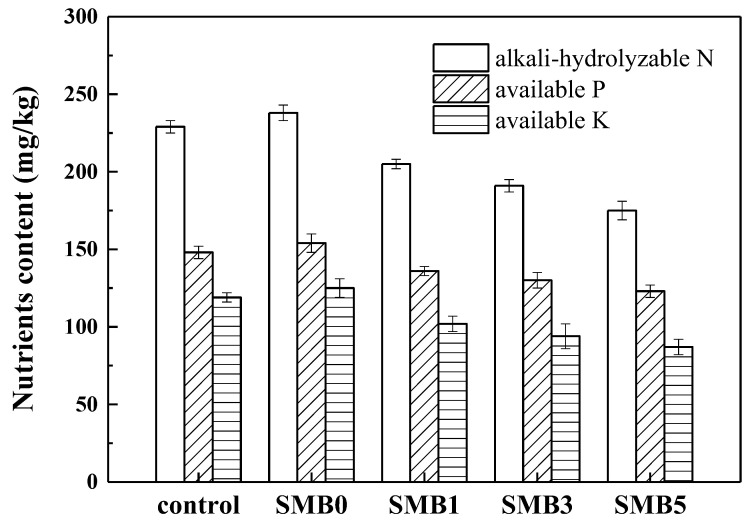
Influence of SMB on soil available nutrients.

The contents of available N, P, and K in soils can particularly influence plant growth and crop productivity [21,37]. The contents of soil alkali-hydrolyzable N, available P, and available K at different SMB proportions were measured and are shown in Figure 4. It can be seen that the soil alkali-hydrolyzable N content gradually decreased from 236 ± 4.58 mg/kg to 175 ± 5.58 mg/kg, available K content decreased from 154 ± 5.32 mg/kg to 123 ± 4.21 mg/kg and available K content decreased from 125 ± 5.16 mg/kg to 88 ± 4.33 mg/kg with the increase of SMB proportions from 0% to 5% (*w*/*w*). Some researchers have also reported the disadvantageous effects of biochar application on soil-available nutrients [38]. The decreased content of available nutrients was mainly explained by the adsorption of nutrients onto the biochar surface, and it was also related to the increased nutrient consumption by plants and microorganisms.

#### 3.3.3. Mechanism of the SMB on Affecting Soil Properties

The co-pyrolysis of sewage sludge and maize straw generated SMB with unique physicochemical properties that synergistically modified soil biogeochemical processes through three interconnected pathways:

(i) The alkaline minerals (e.g., CaCO_3_ and MgO) and soluble cations (K^+^ and Ca^2+^) derived from sewage sludge ash elevated the soil pH (from 6.40 to 7.93) and boosted CEC, facilitating Pb^2+^ immobilization via electrostatic adsorption and hydroxide precipitation.
(ii) Maize straw-derived aromatic carbon structures enhanced soil organic carbon (SOC: 176.79 mg/kg at 5% SMB) while forming microporous networks (pore size: 20–150 nm) that adsorbed nutrients (N, P, K) and stabilized labile organic matter.
(iii) The hybrid mineral-organic matrix of SMB, such as sludge-originated oxides, immobilized Pb through ion exchange, while the induced functional groups (i.e., -COOH, -C-H) promoted microbial enrichment. The prominent C-O stretching band (1033 cm^−1^) from hydroxyl/phenolic groups contributed to elevated soil pH through OH^−^ release, while CEC was via negatively charged surfaces for electrostatic Pb^2+^ adsorption. These groups facilitated Pb immobilization via coordination bonding and phosphate precipitation. The disappearance of maize straw’s C=C (1250 cm^−1^) and sludge’s -CH_2_ (2975 cm^−1^) confirmed carbonization-driven aromaticity, which stabilized soil organic carbon via hydrophobic interactions.

### 3.4. Influence of SMB on Plant Growth

Figure 3d and Figure S1 show the influence of different SMB proportions on ryegrass leaf length and fresh weight after 55 d of incubation. This showed that Pb pollution had a significant impact on ryegrass growth (Figure S1). Compared with the control soil, ryegrass growth in the Pb-contaminated soil (i.e., SMB0) decreased dramatically. After biochar amendment, ryegrass fresh weight and leaf length in contaminated soil improved greatly (0.33 g/pot and 0.89 cm, respectively). When the Pb-contaminated soil was mixed with SMB1, the values of ryegrass weight and leaf length reached 1.41 ± 0.03 g/pot and 7.40 ± 0.04 cm, respectively. However, with the increase in SMB addition proportion from 1% to 5% (*w*/*w*), ryegrass growth in contaminated soil gradually decreased by 0.31 g/pot and 1.20 cm. The increase in ryegrass growth after biochar application could be ascribed to the function of SMB in reducing Pb toxicity and providing ryegrass with nutrients [39]. The results also suggested that a high SMB proportion (e.g., 5%, *w*/*w*) in soil had a negative effect on plant growth, which was mainly ascribed to the decline in soil available nutrients and the increase in salinity with biochar addition [40,41].

### 3.5. Influence of SMB on Microbial Community Structure

**Figure 5:**
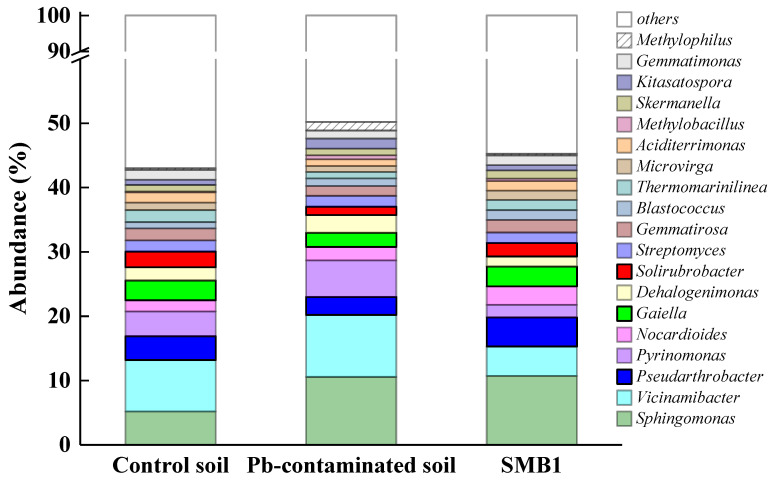
Influence of SMB application on microbial community structure after 55 d of incubation.

In order to investigate the influence of biochar application on microbial community structure and Pb immobilization, bacterial 16S rRNA gene sequences of soil, Pb-contaminated soil (i.e., SMB0), and soil amended with SMB application (1%, *w*/*w*) were analyzed (Figure 5). As shown in Figure 5, the bacterial communities of the three soil types are significantly different. It could be seen that the abundance of the bacterium genus *Sphingomonas*, which was found to be resistant to lead or other heavy metals [42,43], increased dramatically in Pb-contaminated soil and SMB-amended soil compared to the control soil.

The more important change in bacterial communities was that some bacteria with the ability to immobilize Pb were enriched after SMB application in Pb-contaminated soil, mainly including *Pseudarthrobacter*, *Solirubrobacter*, and *Gaiella*. *Pseudarthrobacter* was found in mines and was resistant to heavy metals [44]. *Pseudarthrobacter*, isolated from crude oil-contaminated soils and classified as a non-model strain with bio-safety, can degrade aromatic hydrocarbons and immobilize Pb through biosorption and mineral co-precipitation [45,46]. *Solirubrobacter* belongs to the phylum *Actinobacteria*, which has been reported to have potential heavy metal biosorption ability [47] and could help maintain soil health [48]. Furthermore, studies have reported that *Gaiella* was the dominant bacterium in soil contaminated by heavy metals [49], indicating its good resistance to heavy metals. The improvement in microbial community structure indicated that the biological immobilization of Pb in soil could be enhanced after biochar application.

### 3.6. Influence of SMB on Pb Content in Soil

The influence of different proportions of SMB application on the Pb content in the soil after 55 d of incubation was investigated (Figure S2). The results showed that the Pb content in the soil increased with the increasing SMB addition proportion from 1% to 5% (*w*/*w*). The Pb content in the contaminated soil increased by 33.43% after SMB application compared to that in SMB0, which suggested that SMB can immobilize Pb in soil. Further, the correlation analysis among Pb concentration, soil properties, ryegrass growth, and microbial community structure is presented in Table 2 for deep investigation. Statistical analysis revealed a significant positive correlation (*p* < 0.01) between soil Pb content and both pH and CEC (Table 2). These results align with the established immobilization mechanisms of Pb in biochar-amended soils, predominantly driven by ion exchange (e.g., displacement of Ca^2+^/K^+^ by Pb^2+^), co-precipitation (e.g., formation of PbCO_3_ or Pb(OH)_2_), and surface complexation with oxygen-containing functional groups (e.g., -COOH, -OH), as extensively documented in previous studies [5,9,16]. Researchers have also found that soil properties, such as pH, can increase the immobilization of Pb by providing an alkaline soil environment for better precipitation [16]. Pb immobilization can also be related to soluble P in the soil, which enables Pb absorption.

The Pb immobilization in soil after SMB remediation could be attributed to the following reasons: (i) specific adsorption: the FTIR results showed that the abundant functional groups (e.g., -C-O, -C-N, -P-H) on the SMB surface can form stable materials with Pb through complex functions, leading to the retention of Pb in soil. In addition, the functions of ion exchange with other cations (i.e., K^+^, Ca^2+^, Na^+^, Mg^2+^) were also important for Pb immobilization; (ii) Nonspecific adsorption: it was observed in Figure 4 that soil pH and CEC increased after SMB application, which could contribute to Pb retention in soil by providing a more alkaline environment and exchangeable sites for Pb adsorption. In addition, SOC in the soil and the corresponding microbial activity increased significantly after SMB application, indicating that SOC was important for microbial activity, which could further improve Pb immobilization.

### 3.7. Influence of SMB on Pb Speciation Transformation in Soil

**Figure 6:**
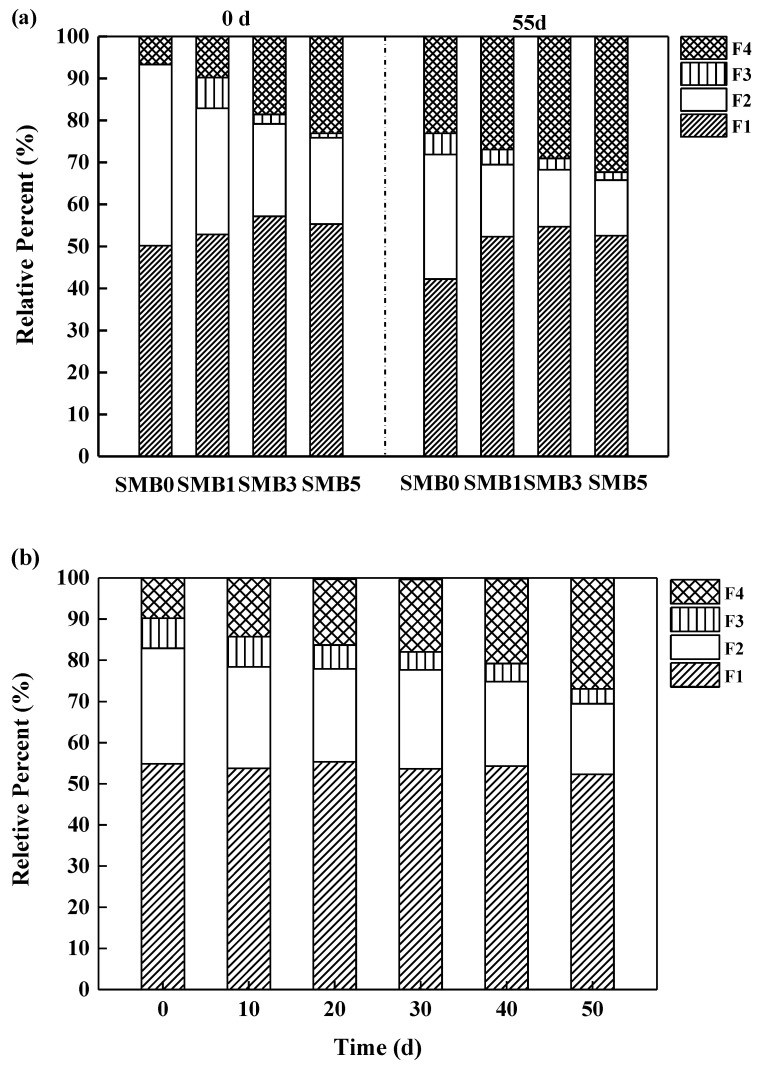
The influence of SMB application on Pb speciation transformation in soil. ((**a**). Pb speciation after different SMB proportions application; (**b**). Pb speciation distribution after SMB1 application during 55 d of incubation).

The modified BCR procedure was applied to evaluate the chemical speciation transformation of Pb with different SMB proportions (Figure 6a). Four types of Pb fractions were identified (Figure 6a): exchangeable fraction, reducible fraction, oxidizable fraction, and residual fraction. The exchangeable fractions (>50%, *w*/*w*) were the main fractions. The percentage of reducible and oxidizable fractions decreased gradually, while the residual fraction increased by about 12% (*w*/*w*) with SMB application on the first day. After 55 d of incubation, the percentage of residual fraction in the contaminated soil significantly increased by 7.3–21.7%, and the exchangeable and reducible fractions declined by 2.7–8.2% and 7.3–11.5%, respectively. In addition, during the 55 d incubation period, the exchangeable fraction gradually decreased from 54.87% to 52.29%, while the residual fraction increased from 9.81% to 26.94% (Figure 6b).

Generally, the sum of the exchangeable fraction and a reducible fraction is used to represent the active heavy metals (i.e., direct toxic metals), while the sum of the residual and oxidable fraction is used to represent the stable fractions [4]. The experimental results indicated that the addition of SMB had a positive effect on Pb stabilization by transforming active metals into stable metal species. The stabilization of soil Pb after SMB amendment could be explained by the comprehensive effect of SMB adsorption and soil properties. The mechanism of exchangeable heavy metal transformation into residual forms may be related to co-precipitation and complex functions with functional groups. The abundant oxygen-containing (e.g., -OH, -C-O) and nitrogen functional groups (i.e., C-N) in SMB (Figure S1) could help biochar bound with Pb to transform into stable fractions. High soil pH can increase the transformation of residual forms by promoting precipitation and complexation.

## 4. Engineering Implications of SMB Application

Based on this research, SMB at different addition proportions (1%, 3%, and 5%, equivalent to 45 t/ha, 136 t/ha, and 227 t/ha, respectively) can be used for heavy metal-contaminated soil remediation. The application of SMB at a higher proportion (5%, *w*/*w*) was more effective in improving soil properties (i.e., organic matters, CEC, and pH values), immobilizing Pb, and transforming its speciation into a stable fraction in the contaminated soil. However, excessive use of biochar might have disadvantageous impacts on nutrient uptake and plant growth. Therefore, this work offers a new opportunity for the co-pyrolysis of sewage sludge and agricultural waste, which is important for countries with incomplete disposal of sewage sludge and large production of agricultural waste. This study provides theoretical support for biochar application and the speciation transformation of heavy metals in contaminated soil.

While pyrolysis offers advantages in waste treatment and energy recovery, its technical limitations and environmental risks require careful evaluation. This process demands substantial energy input, particularly during the moisture removal pretreatment stage, where 20–30% of the total energy consumption may be devoted to drying high-moisture feedstocks [6]. During pyrolysis, incomplete combustion frequently occurs under improper temperature control, generating hazardous byproducts, including carcinogenic polycyclic aromatic hydrocarbons (PAHs) and persistent organic pollutants [50]. These findings highlight the critical need for rigorous emission monitoring systems to mitigate the environmental impact. The remediation effectiveness and cost of pyrolysis should also be considered, which are related to many factors, such as the type of feedstock and properties of sewage sludge.

## 5. Conclusions

The influence of SMB prepared from the pyrolysis of sewage sludge and maize straw on soil properties, plant growth, microbial community structure, and the immobilization and speciation transformation of Pb in the constructed wetland was investigated. It was found that soil pH, CEC, and organic carbon content increased while available nutrient content decreased after SMB application. The highest values of ryegrass fresh weight and leaf length were 1.41 ± 0.03 g/pot and 7.40 ± 0.04 cm, respectively, when the soil was amended with SMB1. In addition, the application of SMB significantly immobilized Pb in the soil with an increase in the SMB proportion, which was mainly related to the complexes with functional groups, high soil pH, and CEC. Pb speciation distribution changed from easily exchangeable and reducible fractions to stable organic-bound fractions in the contaminated soil. Further studies on byproducts of incomplete combustion, energy input, remediation effectiveness, and cost of sewage sludge pyrolysis need to be conducted.

## Figures and Tables

**Table 1:** Physicochemical properties of feedstocks, SMB, and blank sandy soil.

	Sewage Sludge	Maize Straw	SMB	Sandy Soil	Unit
Moisture	2.25	4.59	2.33	22.11	wt%
Ash	29.53	1.69	54.44	–	wt%
VM ^a^	61.52	78.08	14.65	–	wt%
FC ^b^	6.70	15.64	28.58	–	wt%
C	53.21	49.20	21.18	1.21	wt%
H	7.53	6.31	2.73	0.91	wt%
O	30.89	43.74	73.51	50.34	wt%
N	6.39	0.45	1.48	0.12	wt%
pH	5.9–6.0	–	8.1–8.2	6.3–6.4	–
CEC ^c^	–	–	29.93 ± 0.55	17.32 ± 0.13	cmol/kg
Surface area	–	–	163.11	–	m^2^/g
Pb	5.04 ± 0.79	8.75 ± 0.02	32.56 ± 0.41	17.83 ± 0.62	mg/kg
Cd	ND ^d^	ND ^d^	0.06 ± 0.01	0.09 ± 0.15	mg/kg
Mg	0.63 ± 0.15	3.30 ± 0.02	4.83 ± 0.46	4.56 ± 1.31	g/kg
Na	0.38 ± 0.08	0.90 ± 0.09	1.01 ± 0.08	0.18 ± 0.06	g/kg
K	0.49 ± 0.09	11.87 ± 1.30	5.09 ± 0.07	0.88 ± 0.15	g/kg
Al	8.55 ± 0.64	3.62 ± 1.04	35.26 ± 0.67	4.26 ± 0.41	g/kg
Cu	0.05 ± 0.02	0.04 ± 0.02	0.21 ± 0.09	0.02 ± 0.10	g/kg
Fe	2.32 ± 0.23	3.45 ± 1.42	20.76 ± 0.21	9.06 ± 0.79	g/kg
Mn	0.04 ± 0.01	0.12 ± 0.20	0.33 ± 0.07	0.24 ± 0.09	g/kg
Zn	0.28 ± 0.05	0.18 ± 0.03	1.35 ± 0.03	0.08 ± 0.10	g/kg
Ca	7.38 ± 0.21	15.32 ± 1.26	44.11 ± 0.26	15.80 ± 1.94	g/kg

^a^ volatile matter; ^b^ fixed carbon; ^c^ cation-exchange capacity; ^d^ not detected.

**Table 2:** Correlation analysis of soil properties, ryegrass growth, microbial community, and Pb concentration after 55 d of incubation.

	SMB Proportion	pH	SOC	CEC	Ryegrass Weight	Leaf Length	Microbial Community
pH	0.818						
SOC	0.987 *	0.794					
CEC	0.898	0.978 *	0.856				
Ryegrass weight	−0.010	0.559	−0.009	0.389			
Leaf length	−0.450	0.125	−0.427	−0.071	0.891		
Microbial community	−0.300	−0.074	−0.446	−0.032	0.151	0.169	
Soil Pb	0.812	0.997 **	0.775	0.983 *	0.551	0.113	−0.002

* = *p* < 0.05, ** = *p* < 0.01.

## Data Availability

The original contributions presented in this study are included in this article. Further inquiries should be directed to the corresponding author.

## Supplementary Materials

The following supporting information can be downloaded at: https://www.mdpi.com/article/10.3390/biology14050515/s1, Figure S1: Ryegrass growth with different SMB proportions after 55 d incubation; Figure S2: Influence of SMB application on Pb concentration in soil after 55 d incubation; Table S1: Leaching heavy metal contents (mg/kg) in SMB.
