# Supplementary material for: Application of Biochar on Soil Improvement and Speciation Transformation of Heavy Metal in Constructed Wetland
Source: Biology (Basel). 2025 May 7;14(5):515. doi: 10.3390/biology14050515 (PMC12108646; doi:10.3390/biology14050515)
Supplement: Supplementary file 1 [file biology-14-00515-s001.zip › biology-3574671-supplementary.pdf]

Supplementary material of

Application of Biochar on Soil Improvement and Speciation Transformation of Heavy Metal in Constructed Wetland

Yuan Zhou [1,2], Xiaoqin Nie [1,2,*], Yao Zhao [3], Liqiu Zhang [4,5,*], Yatian Cheng [1,2,6], Cancan Jiang [7], Wenbin Zhao [1,2], Xiangchun Wang [1,2] and Chao Yang [1,2]

Summary

Figure S1. Ryegrass growth with different SMB proportions after 55 d incubation.

Figure S2. Influence of SMB application on Pb concentration in soil after 55 d incubation.

Table S1. Leaching heavy metal contents (mg/kg) in SMB.

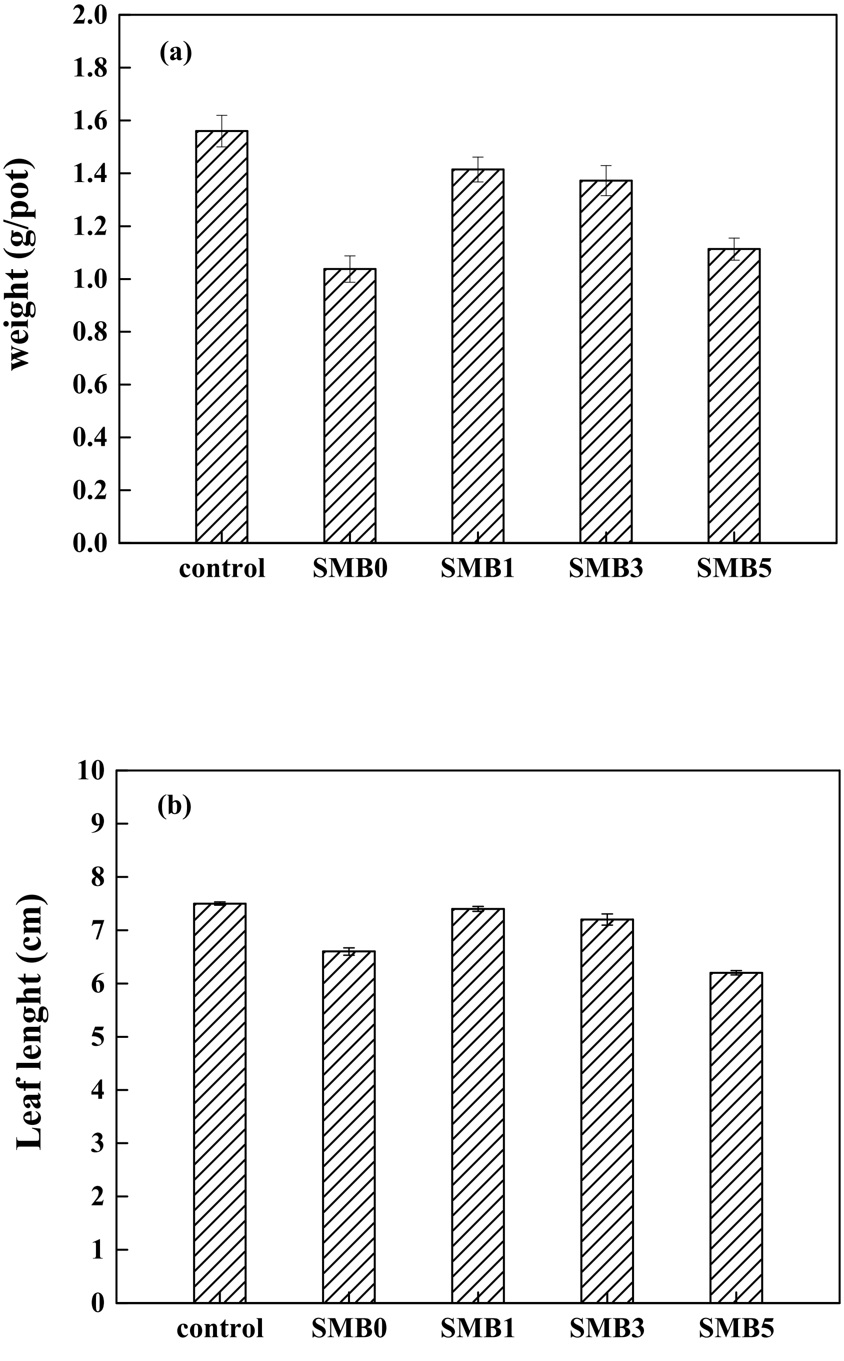

Figure S1. Ryegrass growth with different SMB proportions after 55 d incubation.

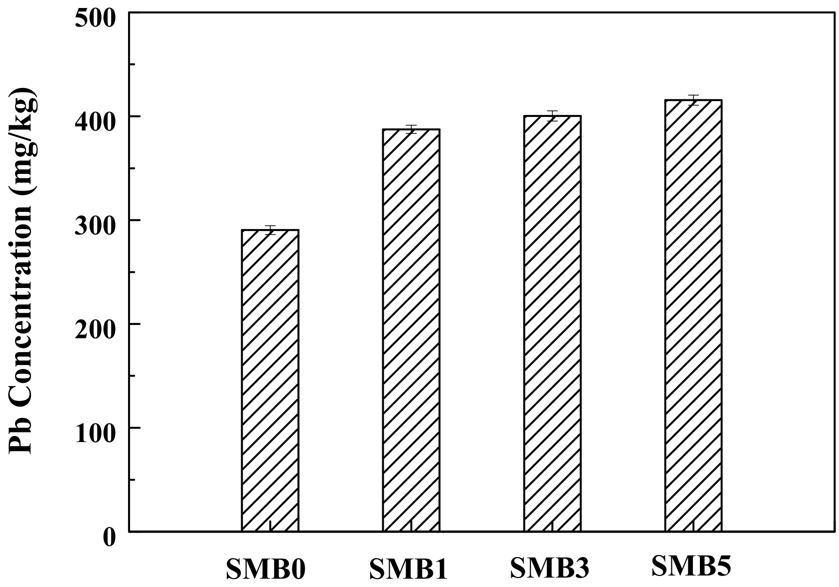

Figure S2. Influence of SMB application on Pb concentration in soil after 55 d incubation.

Table S1. Leaching heavy metal contents (mg/kg) in SMB.

	Zn	Cu	Pb	Cr	Cd	Ni
Value	75~80	10±0.30	0.2±0.10	0.1±0.10	—	—
GB 5085.3-2007	100	100	5	5	1	5
